# Sema3A Antibody BI-X Prevents Cell Permeability and Cytoskeletal Collapse in HRMECs and Increases Tip Cell Density in Mouse Oxygen-Induced Retinopathy

**DOI:** 10.1167/tvst.11.6.17

**Published:** 2022-06-21

**Authors:** Nina Zippel, Cynthia Hess Kenny, Helen Wu, Michel Garneau, Rachel Kroe-Barrett, Priyanka Gupta, Sarah Low, Remko A. Bakker, Leo Thomas

**Affiliations:** 1 Boehringer Ingelheim Pharma GmbH & Co. KG, Biberach, Germany; 2 Boehringer Ingelheim Pharmaceuticals Inc., Ridgefield, CT, USA

**Keywords:** avascular area, diabetic macular ischemia, diabetic retinopathy, semaphorin 3A, tip cell density

## Abstract

**Purpose:**

Semaphorin 3A (Sema3A) is an axonal guidance molecule that inhibits angiogenesis by vasorepulsion and blocks revascularization in the ischemic retina. BI-X is an intravitreal anti-Sema3A agent under clinical investigation in patients with proliferative diabetic retinopathy (PDR) and diabetic macular ischemia (DMI).

**Methods:**

Surface plasmon resonance was used to determine binding affinity of BI-X to human and murine Sema3A. In vitro, human retinal microvascular endothelial cells (HRMECs) were used to assess effects of BI-X on cell permeability and cytoskeletal collapse induced by Sema3A. In vivo, intravitreal BI-X or an anti-trinitrophenol control antibody was administered in both eyes in mice with oxygen-induced retinopathy (OIR). Retinal flat mounts were prepared, and avascular area and tip cell density were determined using confocal laser-scanning microscopy.

**Results:**

Dissociation constants for BI-X binding to human and murine Sema3A were 29 pM and 27 pM, respectively. In vitro, BI-X prevented HRMEC permeability and cytoskeletal collapse induced by Sema3A. In vivo, BI-X increased tip cell density by 33% (*P* < 0.001) and reduced avascular area by 12% (not significant). A significant negative correlation was evident between avascular area and tip cell density (*r*^2^ = 0.4205, *P* < 0.0001).

**Conclusions:**

BI-X binds to human Sema3A with picomolar affinity and prevents cell permeability and cytoskeletal collapse in HRMECs. BI-X also enhances revascularization in mice with OIR.

**Translational Relevance:**

BI-X is a potent inhibitor of human Sema3A that improves revascularization in a murine model of OIR; BI-X is currently being investigated in patients with laser-treated PDR and DMI.

## Introduction

Diabetic retinopathy (DR) is the leading cause of preventable vision loss in adults of working age.^1^ Depending on imaging modality and definition, up to 77% of patients with DR also have diabetic macular ischemia (DMI), which is central to the severe stages of DR and is therefore highly prevalent in individuals with proliferative DR, the most severe form of the disease.^2^^,^^3^ DMI and diabetic macular edema (DME) also often coexist in patients with DR.^4^

The principal feature of DMI is enlargement of the foveal avascular zone (FAZ), along with reduced mean retinal arteriolar diameter and loss of the macular capillary network (capillary dropout).^5^^–^^7^ An enlarged FAZ correlates with reduced visual acuity^8^; therefore retinal damage caused by DMI can result in progressive and marked vision impairment.^2^^,^^9^^,^^10^ Despite the major clinical burden of DMI, no treatments are currently approved or in clinical development specifically for the management of patients with the disease.^2^^,^^9^ In addition, DMI may have a detrimental influence on the efficacy of anti-vascular endothelial growth factor-A (VEGF-A) therapies for DME,^11^ highlighting the urgent unmet clinical need for DMI-specific treatments.

**Figure 1.**
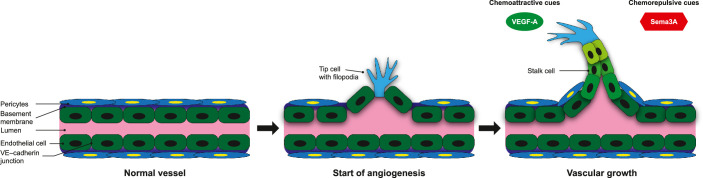
Diagrammatic representation of sprouting angiogenesis in the retina, adapted from Sapieha.^17^ Initially, tip cell filopodia are stimulated by proangiogenic factors, such as VEGF-A. Via a combination of pericyte detachment from the vascular basement membrane, matrix metalloproteinase-induced degradation of the basement membrane, and loosening of VE–cadherin junctions, tip cell filopodia migrate through the basement membrane and search for vascular guidance cues.^12^ Chemoattractive cues include VEGF-A, whereas chemorepulsive cues include Sema3A.^13^^,^^14^ Subsequently, endothelial cells adjacent to the tip cells (i.e., “stalk” cells) proliferate in response to proangiogenic factors (e.g., VEGF-A) to elongate the neovessels.^13^^–^^16^ Sema3A, semaphorin 3A; VE, vascular endothelial; VEGF-A, vascular endothelial growth factor-A.

Through VEGF receptor-2 and neuropilin-1 (Nrp-1) signaling, VEGF-A is the primary driver of angiogenesis.^12^^,^^13^ In sprouting angiogenesis, specialized endothelial “tip” cells guide newly forming vessels by extending long filopodia with a high density of growth factor receptors, thereby enabling the detection of growth factor gradients and migration by vasoattraction or vasorepulsion.^13^^,^^14^ VEGF-A is a well-established proangiogenic factor that not only attracts migrating tip cells but also promotes the subsequent proliferation of adjacent “stalk” cells, leading to elongation of newly forming vessels.^13^^,^^14^ Thus a principal factor regulating angiogenesis is the balance between tip cell and stalk cell activity (Fig. 1).^12^^–^^17^

Overall, the effects of angiogenesis-promoting factors on endothelial tip cells have been extensively investigated, whereas the effects of angiogenesis-inhibiting, ­repelling, or -redirecting factors have been relatively poorly defined.^13^ Nonetheless, class 3 semaphorins and their resulting plexin and neuropilin receptor complexes have important functions in vascular guidance,^18^^,^^19^ and disruption of murine Nrp-1–semaphorin 3 signaling leads to dysregulated vascular patterning.^20^

Key among the class 3 semaphorins is semaphorin 3A (Sema3A), an axonal guidance molecule that inhibits angiogenesis by vasorepulsion.^21^^–^^23^ Experimental animal studies have shown that, in the eye, Sema3A is involved in vascular degeneration by blocking physiologic revascularization in the ischemic retina.^23^^–^^25^ Sema3A is secreted by retinal ganglion cells under hypoxic conditions and binds to Nrp-1 and plexin-A, thereby inducing cytoskeletal collapse in the filopodia of endothelial tip cells in newly forming retinal vessels.^24^ Thus Sema3A forms a chemical barrier that repels new blood vessels away from the ischemic retina and towards the vitreous.^24^ In a murine model of oxygen-induced retinopathy (OIR), silencing *Sema3A* gene expression enhances physiologic vascular regeneration in the ischemic retina, thus inhibiting destructive neovascularization and preserving neuroretinal function.^24^ Sema3A is therefore thought to play a key role in the repeating cycle of capillary and neuronal damage that leads to gradual vision loss in DMI.^9^ In addition, raised Sema3A levels have been found in the vitreous and plasma of patients with proliferative DR and/or DME.^26^^,^^27^

BI-X is an intravitreal anti-Sema3A agent under clinical investigation in patients with laser­treated proliferative DR and DMI. BI-X-induced neutralization of the chemical barrier created by Sema3A is expected to improve physiologic revascularization of ischemic areas and thus restore oxygen and nutrient supply, thereby protecting the neuroretina and improving retinal function.^24^

In this article, we outline the preclinical data on the binding kinetics of BI-X, and highlight the functional effects of BI-X on cell permeability and cytoskeletal collapse in vitro*,* and on tip cell density and avascular area in a murine model of OIR.

## Methods

### Binding Kinetics of BI-X

#### Materials

EDC (1-ethyl-3-[3-dimethylaminopropyl] carbodiimide hydrochloride) and NHS (sulfo-N-hydroxysulfosuccinimide) for amine coupling to the surface were obtained from Bio-Rad Laboratories (Hercules, CA, USA). Tween 20 (Sigma-Aldrich, St. Louis, MO, USA) was added to phosphate-buffered saline solution–Tween–ethylenediamine tetra-acetic acid (PBS-T-EDTA; Bio-Rad Laboratories) to make the final concentration of the Tween 20 running buffer 0.01%. Human Sema3A (accession no. Q14563) containing the semaphorin and plexins-semaphorins-integrins domains with an N-terminal histidine tag was produced in house, based on the design by Antipenko et al.^28^ Mouse Sema3A (accession no. O08665) was produced in house with a similar design. All proteins were expressed in HEK293-6E cells (National Research Council Canada, Ottawa, ON, Canada), and purification was performed by Ni-NTA chromatography (Qiagen, Beverly, MD, USA) followed by gel filtration chromatography using Superdex 200 resin (Cytiva, Marlborough, MA, USA).

#### Surface Plasmon Resonance Binding Method

Surface plasmon resonance studies were performed on a ProteOn XPR36 system (Bio-Rad Laboratories). The running buffer was PBS-T-EDTA with 0.01% Tween 20. Human fragment-antigen binding binder (GE Healthcare, Chicago, IL, USA) was amine coupled to the surface of a GLM sensor chip (Bio-Rad Laboratories). BI-X (0.5 µg/mL) was captured on the surface. Twofold dilutions (starting at 10 nM) of Sema3A were injected over the surface. The resulting sensorgrams were fitted globally to 1:1 Langmuir binding (ProteOn Manager, version 3.1.0.6, Bio-Rad Laboratories) to provide values for on-rate (*k*_a_), off-rate (*k*_d_), and dissociation constant (*K*_D_).

### Functional Effects of BI-X in an In Vitro Permeability Assay

A Millipore ECM642 (96-well) in vitro vascular permeability transwell assay kit (Merck KGaA, Darmstadt, Germany) was used; cell culture inserts in the kit wells contain 1 µm pores coated with type I rat tail collagen. Human retinal microvascular endothelial cells (HRMECs; Applied Cell Biology Research Institute 181; Cell Systems, Kirkland, WA, USA) were seeded at a density of 25,000 cells/well onto the cell culture inserts and allowed to grow into a monolayer for 72 hours. The HRMECs were treated overnight with either 100 ng/mL recombinant human (rh) VEGF-A (293-VE010; R&D Systems, Inc., Minneapolis, MN, USA) or 500 ng/mL rh-Sema3A (1250-S3; R&D Systems) with and without 1 µg/mL of BI-X or 1 µg/mL of anti-trinitrophenol (TNP; 1B7.11 [ATCC TIB-191]; American Type Culture Collection, Manassas, VA, USA). For each group, eight wells were treated per experiment.

In vitro cell permeability was determined via increased passage of high-molecular-weight fluorescein isothiocyanate (FITC)-dextran through confluent layers of HRMECs for 60 minutes using fluorescence measurements at 485 and 535 nm with a SpectraMax M5 microplate reader (Molecular Devices, San Jose, CA, USA). Fluorescence values of blank medium were deducted from actual experimental values and data were normalized to the control group. Prism software (version 7.0; GraphPad Software, San Diego, CA, USA) was used to present all values as mean ± standard error of the mean (SEM). Multiple between-group comparisons were conducted using one-way analysis of variance and Dunnett's post hoc test for in vitro permeability values relative to Sema3A-treated HRMECs.

### Functional Effects of BI-X in a Semaphorin-Induced In Vitro Cytoskeletal Collapse Assay

The functional potency of BI-X was determined in a Sema3A-induced cytoskeletal collapse assay in HRMECs. The collapse was measured as a reduction in cellular impedance using the xCELLigence System (Agilent Technologies, Santa Clara, CA, USA). HRMECs were seeded in complete medium (4Z0-500; Cell Systems) at a density of 20,000 cells/well and incubated overnight under normal growth conditions in the xCELLigence System; the cell index was measured every hour. During this time period, cells were grown to confluency, indicated by the cell index reaching a plateau. HRMECs were stimulated with rh-Sema3A (1250-S3; R&D Systems) to determine concentration–response curves, with and without four different concentrations of BI-X in the presence of 3 mM calcium chloride, and the cell index was measured every 10 minutes over a stimulation period of five hours; data were recorded as mean ± SEM.

Potency was determined by the ability of BI-X to shift the concentration–-response curve (i.e., the 50% inhibitory concentration [IC_50_]) of rh-Sema3A, using Prism software version 7.0 (GraphPad Software) Gaddum Schild IC_50_ shift equations, and was calculated from the pA_2_ value, as potency = 10^−^*^x^*, where *x* is the pA_2_ value. For specificity determination, HRMECs were seeded in complete medium at a density of 20,000 cells/well and incubated overnight under normal growth conditions in the xCELLigence System; the cell index was measured every hour.

HRMECs were stimulated with 500 ng/mL rh-Sema3A (1250-S3; R&D Systems), rh­Sema3C (5570-S3; R&D Systems), or rh-Sema3E (3239-S3 [now replaced by 3239-S3B]; R&D Systems), or 2 µg/mL recombinant mouse (rm) Sema3B (5440-S3; R&D Systems) or rm-Sema3F (3237-S3; R&D Systems), with and without 2 µg/mL BI-X. The stimulation medium was added to the HRMECs with 3 mM calcium chloride and the cell index was measured every 10 minutes over a stimulation period of two hours. For each group, four wells were treated per experiment. Data were recorded as mean ± SEM.

### Effects of BI-X on Tip Cell Density and Avascular Area in a Murine Model of OIR

Pregnant C57BL/6J mice with a specified mating date were obtained from Charles River Laboratories (Sulzfeld, Germany). Dams and their pups were single-housed, and on postnatal day (P) 6, the mice were stratified by litter size and body weight. Experimental protocols regarding the use of laboratory animals were reviewed by a Federal Ethics Committee and approved by government authorities, and the study adhered to the Association for Research in Vision and Ophthalmology Statement for the Use of Animals in Ophthalmic and Vision Research.

From P7 to P12, dams and their pups were exposed to 75% oxygen in oxygen chambers (BioSpherix, Parish, NY, USA); oxygen content in the chambers was regulated by ProOx P110 controllers (BioSpherix). The mice were returned to normoxia at P12 and pups with a body weight ≥4.5 g (n = 23) received an intravitreal injection of 10 µg of either BI-X or an anti-TNP control antibody in both eyes. The anti-TNP antibody has the same format as BI-X, but targets an epitope (trinitrophenol) that is not present in vivo. Before intravitreal injection, the pups were anesthetized with isoflurane 3% v/v (CP-Pharma Handelsgesellschaft mbH, Burgdorf, Germany) and treated with analgesic eye drops (Novesine 0.4% w/v; OmniVision GmbH, Puchheim, Germany). Injection into the vitreous was performed with a 34-gauge Hamilton syringe (Fisher Scientific GmbH, Schwerte, Germany) at the ora serrata, and, after injection, the eyes were moistened with GenTeal eye drops (Alcon Pharma GmbH, Freiburg, Germany).

At P17, the mice were sacrificed by cervical dislocation under isoflurane anesthesia, the eyes were enucleated, and retinal flat mounts were prepared. The eyes were fixed in paraformaldehyde 4% (Boster Biological Technology, Pleasanton, CA, USA) for one hour at 4°C and then transferred to PBS. The eyes were cut along the ora serrata, and the cornea, iris, ciliary body, and lens were removed. The retinae were separated from the outer ocular segments (sclera and choroidea) and stained with FITC-labeled isolectin B4 (no. L9381; Sigma-Aldrich). Retinae were incubated overnight at 4°C with 1% bovine serum albumin and 0.5% Triton X-100 (SERVA Electrophoresis GmbH, Heidelberg, Germany) in PBS, and washed with 1 mM calcium chloride, 1 mM magnesium chloride, and 1 mM manganese chloride in PBS. Staining was performed with 0.02 mg/mL FITC-isolectin B4 overnight, in the dark, at 4°C. Retinae were then washed with PBS, fixed for another five minutes with paraformaldehyde, and washed again. Each retina was transferred to a glass slide and cut four times to achieve a flat cloverleaf-like structure. The tissue was covered with mounting medium and a coverslip was placed on top to obtain a retinal flat mount. The flat mounts were stored in the dark at 4°C until analysis.

To determine the avascular area and tip cell density, the retinal flat mounts underwent excitation at a wavelength of 488 nm with an LSM 700 confocal laser-scanning microscope (Carl Zeiss AG, Oberkochen, Germany) and retinal images were obtained. Total and avascular retinal areas were calculated with the instrument program ZEN (blue edition; Carl Zeiss AG). Avascular area was expressed as a percentage of total retinal area. After retinal images were opened in the instrument program ImageJ (Carl Zeiss AG), tip cells at the avascular front were manually counted by an observer masked to the experimental group. Tip cell density was calculated as the number of tip cells at the avascular front normalized to the length of the avascular front, which was measured by delineation in the ImageJ program. Data were analyzed by two-sided, unpaired *t*-test using Prism software version 7.0 (GraphPad Software). The mean value for the left and right eye of each mouse pup was used for statistical analysis.

## Results

### BI-X Binds to Human Sema3A in an Antibody-Capture Assay

Results from our assay experiments show that the *k*_a_ values for BI-X binding to human and murine Sema3A are 2.8 and 3.2 × 10^6^ M^–1^ s^–1^, respectively. *k*_d_ values for BI-X binding to human and murine Sema3A are 8.1 and 8.5 × 10^–5^ s^–1^, respectively. *K*_D_ values for BI-X binding to human and murine Sema3A are 29 pM and 27 pM, respectively.

### BI-X Completely Prevents Sema3A-Induced, but not VEGF-A-Induced, Cell Permeability in HRMEC Assays

**Figure 2.**
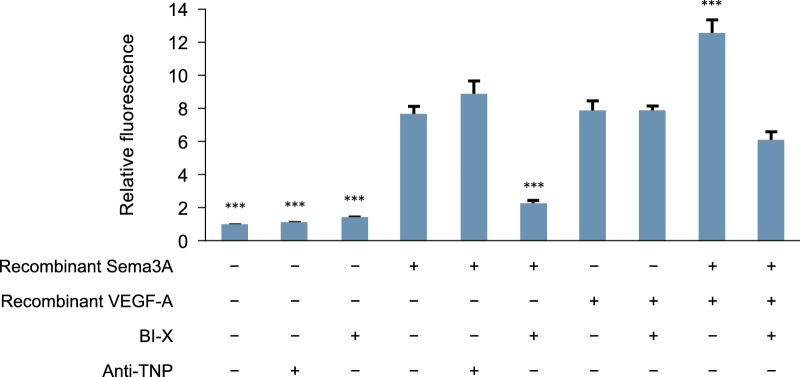
Effects of BI-X, Sema3A, and VEGF-A on HRMEC permeability in vitro. Data are presented as mean ± SEM (group size, eight wells/group) and represent one of two independent experiments with comparable results. Statistical analysis was performed by one-way analysis of variance with Dunnett's post hoc multiple comparisons test versus Sema3A (***P < 0.001). HRMEC, human retinal microvascular endothelial cell; SEM, standard error of the mean; Sema3A, semaphorin 3A; TNP, trinitrophenol; VEGF-A, vascular endothelial growth factor-A.

Human Sema3A and human VEGF-A each increased HRMEC permeability, and the combination of both resulted in a greater increase than that with each factor alone. BI-X completely prevented HRMEC permeability induced by Sema3A but not that induced by VEGF-A (Fig. 2), suggesting distinct effects of each factor on HRMEC permeability.

### BI-X Specifically Prevents Sema3A-Induced Cytoskeletal Collapse in HRMECs

**Figure 3.**
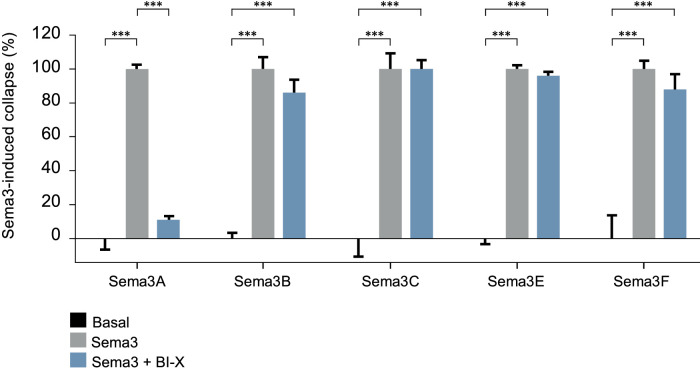
Specificity of BI-X in preventing cytoskeletal collapse induced by Sema3A rather than collapse induced by other class 3 semaphorins. Data are presented as mean ± SEM (group size, four wells/group) Statistical analysis was performed with Tukey's multiple comparisons test (*** *P* < 0.001). SEM, standard error of the mean; Sema3A–F, semaphorin 3–F.

BI-X prevented cytoskeletal collapse induced by rh-Sema3A in HRMECs with a potency of 69 pM (pA_2_ 10.16). Based on values for the cell index (Fig. 3; Supplementary Fig. S1), the mean relative effect size for BI­X plus rh-Sema3A versus Sema3A alone was 11.6%, indicating that BI­X almost completely prevented rh-Sema3A-induced cytoskeletal collapse in HRMECs. BI-X did not prevent cytoskeletal collapse induced by rm-Sema3B, rh-Sema3C, rh­Sema3E, or rm-Sema3F (Fig. 3). Based on values for the cell index, the mean relative effect sizes for BI-X plus semaphorin versus the corresponding semaphorin alone were 87.0% (BI-X plus rm-Sema3B vs Sema3B alone), 100.1% (BI-X plus rh­Sema3C), 97.0% (BI-X plus rh-Sema3E), and 88.0% (BI-X plus rm-Sema3F) (Fig. 3). Cytoskeletal collapse over time can be seen in Supplementary Figure S2.

### BI-X Increases Tip Cell Density and Reduces Ischemic Avascular Area in a Murine Model of OIR

**Figure 4.**
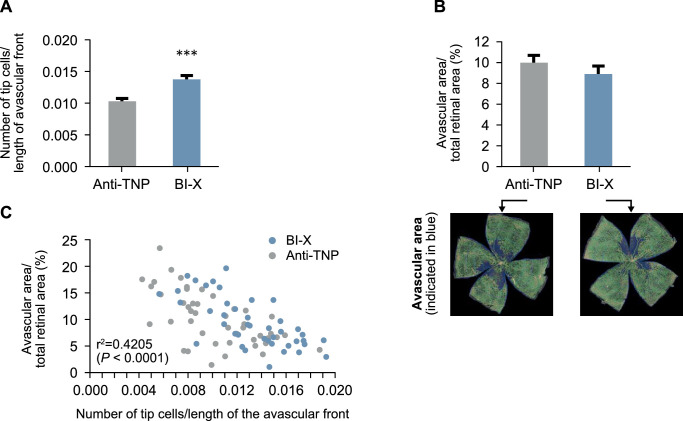
Tip cell density (A), avascular area (B), and their correlation (C) in retinal flat mounts from mouse pups on postnatal Day 17. Data for tip cell density and avascular area are presented as mean ± SEM for average of the right and left eye (n* *= 23 pups; statistical analysis was performed by two-sided, unpaired *t*-test [***P < 0.001]). Data for the correlation are presented for each individual eye (n = 45; Pearson correlation analysis yielded *r*^2^ = 0.4205 [two-sided *P* < 0.0001]). SEM, standard error of the mean; TNP, trinitrophenol.

Tip cell density was significantly increased by 33% (*P* < 0.001) in eyes treated with BI-X compared with the anti-TNP control antibody (Fig. 4A). An example of tip cell morphology is shown in Supplementary Figure S3. Furthermore, avascular area was reduced by 12% (not statistically significant) in eyes treated with BI-X compared with the anti-TNP control antibody (Fig. 4B). A significant negative correlation was evident between avascular area and tip cell density (*r*^2^ = 0.4205, *P* < 0.0001; Fig. 4C).

## Discussion

Modulation of Sema3A in the eye is a promising innovative approach to treating DMI. Previous experimental animal studies have revealed that Sema3A impedes physiological revascularization in ischemic regions in the retina. Sema3A creates a local chemical barrier that repels endothelial tip cells by causing cytoskeletal collapse in their filopodia, thus misdirecting new vessels toward the vitreous and away from ischemic areas.^23^^,^^24^ Moreover, silencing *Sema3A* gene expression in a murine model of OIR maintains neuroretinal function,^24^ suggesting that Sema3A may play a deleterious pathological role during retinal ischemia and in human DMI by contributing to capillary damage and subsequent neuronal malnutrition, build-up of waste products, and injury, resulting in progressive vision loss.^9^

Our preclinical studies confirm that BI-X has a high affinity for human and murine Sema3A and potently and specifically prevents Sema3A function. BI-X administered intravitreally into the murine eye significantly increases tip cell density at the avascular front between vascularized and nonvascularized retinal regions and reduces the size of the avascular area. A negative correlation between tip cell density and avascular area indicates that increased numbers of tip cells promote the growth of new vessels in ischemic areas. The correlation reinforces the fact that increased tip cell density is a prerequisite for revascularization of the avascular area. Furthermore, the avascular area was smaller overall in the retina of eyes that had been treated with BI-X than in control eyes, resulting in a numerical reduction in this in vivo model. The important implication of this finding, which warrants further investigation, is that in the clinical setting of DMI management, increased tip cell density and filopodial extension are likely prerequisites for FAZ revascularization. Interestingly, besides its deleterious effects on vascular guidance, Sema3A is a driver of vascular permeability, like VEGF-A. These results also highlight a potentially important pharmacological distinction between BI-X and anti-VEGF agents, because BI-X was shown to prevent Sema3A-induced, but not VEGF-A-induced, cell permeability in HRMEC assays.

In the clinical setting, anti-VEGF therapies are widely used for the management of DME but may exacerbate existing retinal ischemia by reducing angiogenesis and revascularization.^11^ DMI may also have a detrimental effect on the efficacy of anti-VEGF therapies for DME, highlighting the importance of novel therapies that are unrelated to the VEGF-A pathway.^11^ BI-X is currently under investigation in patients with laser-treated proliferative DR and DMI in the ongoing Phase I/IIa HORNBILL study.^29^ This particular patient population was selected because, among patients with DR, those patients with proliferative DR treated with pan-retinal photocoagulation are likely to have the most severe macular ischemia and the greatest central vision loss.^2^^,^^30^

In summary, our preclinical studies confirm that BI-X binds to human Sema3A with picomolar affinity, potently and specifically inhibiting Sema3A function and preventing cell permeability and cytoskeletal collapse in HRMECs in vitro. BI-X also enhances revascularization in a murine model of OIR. Given these early findings with BI-X, this humanized Sema3A antibody warrants further investigation.

## Supplementary Material

Supplement 1

Supplement 2

Supplement 3
