# Supplementary material for: Sema3A Antibody BI-X Prevents Cell Permeability and Cytoskeletal Collapse in HRMECs and Increases Tip Cell Density in Mouse Oxygen-Induced Retinopathy
Source: Transl Vis Sci Technol. 2022 Jun 21;11(6):17. doi: 10.1167/tvst.11.6.17 (PMC9233289; doi:10.1167/tvst.11.6.17)

Supplementary Figure 1.
Specificity of BI-X in preventing cytoskeletal collapse induced by Sema3A rather than collapse induced by other class 3 semaphorins. Data are presented as normalized cell indices. Group size was n=4 wells/group.
Sema3, Semaphorin 3.

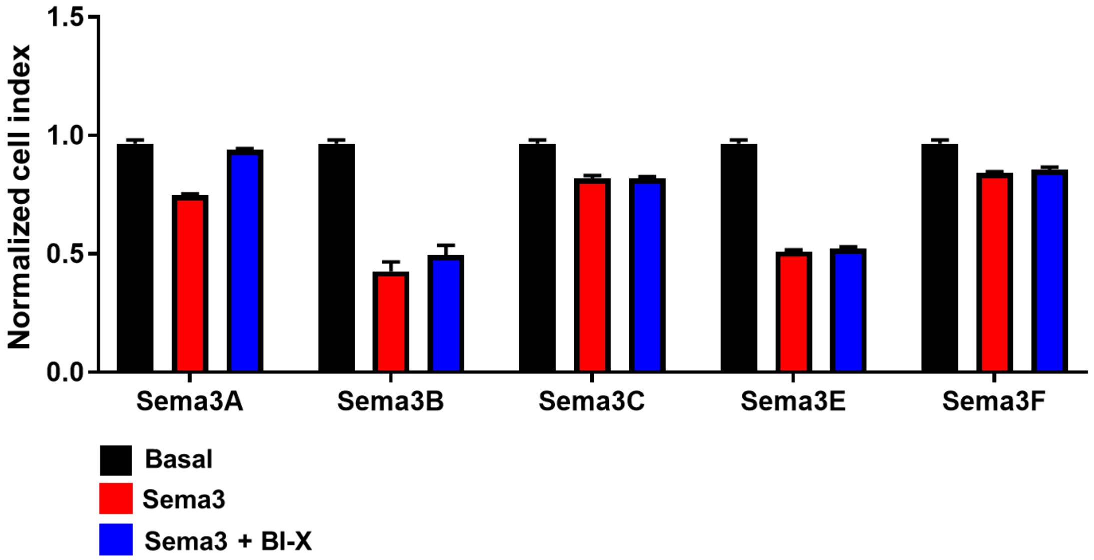

Supplement: Supplement 1 [file tvst-11-6-17_s001.pdf]
