# Supplementary material for: Sema3A Antibody BI-X Prevents Cell Permeability and Cytoskeletal Collapse in HRMECs and Increases Tip Cell Density in Mouse Oxygen-Induced Retinopathy
Source: Transl Vis Sci Technol. 2022 Jun 21;11(6):17. doi: 10.1167/tvst.11.6.17 (PMC9233289; doi:10.1167/tvst.11.6.17)

Supplementary Figure 3.

Tip cell quantification at the revascularization front.

(A) Example of a tip cell at high magnification. Bar 10 µm.

(B) Representative flat mount stained with Isolectin B4 (left, bar 1000 µm) enlarged to show the revascularization front with tip cells (right).

A

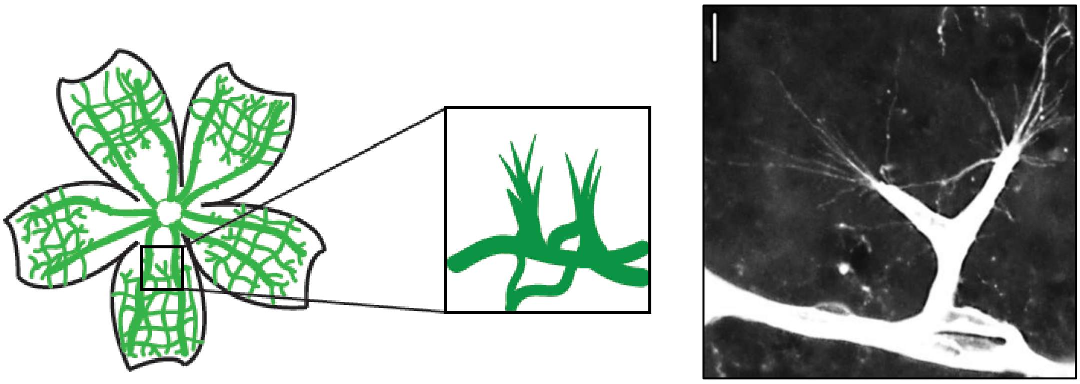

B

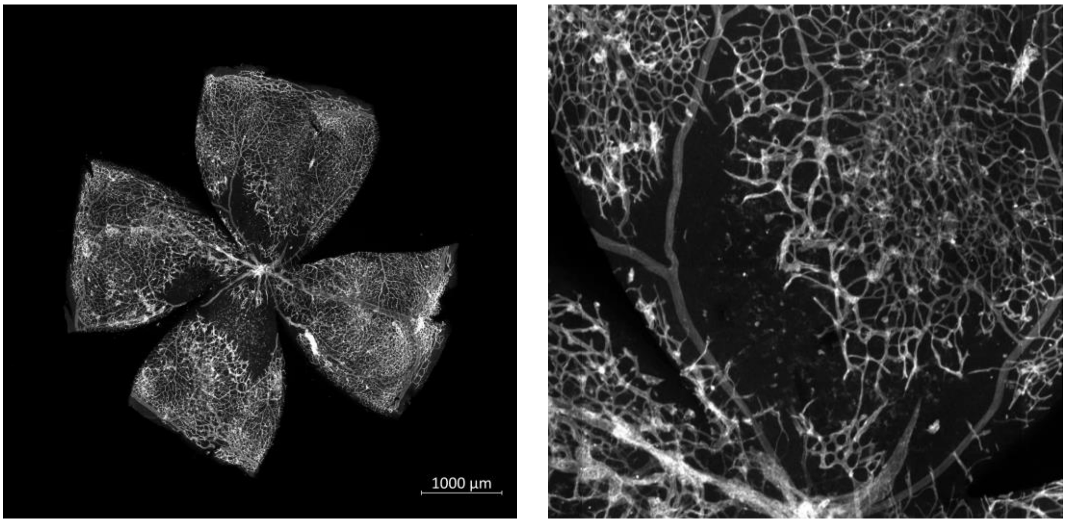

Supplement: Supplement 3 [file tvst-11-6-17_s003.pdf]
